# Antitumor *Trans* Platinum Adducts of *GMP* and *AMP*

**DOI:** 10.1155/MBD.2000.169

**Published:** 2000

**Authors:** Yangzhong Liu, Maria F. Sivo, Giovanni Natile, Einar Sletten

**Affiliations:** 1 Department of Chemistry University of Bergen Allegt.41 Bergen N-5007 Norway; 2 Department of Pharmaceutical Chemistry University of Bari Via E. Orabona 4 Bari I-70125 Italy

## Abstract

Recently it has been shown that several analogues of the clinically ineffective *trans*-DDP exhibit antitumor activity comparable to that of *cis*-DDP. The present paper describes the binding of antitumor *trans*-[PtCl_2_(*E*-iminoether)_2_] (*trans-EE*) to guanosinemonophosphate (GMP) and adenosinemonophosphate (AMP). We have used HPLC and ^1^H and ^15^N NMR to characterize the different adducts. In the case of a 1:1 mixture of *trans-EE* and GMP, at an early stage of the reaction, a monofunctional adduct is formed which, subsequently, is partly converted into a monosolvated monofunctional species. After about 70 hours an equilibrium is established between chloro and solvato monofunctional adducts at a ratio of 30/70. In the presence of excess GMP (4:1) the initially formed monofunctional adducts react further to give two bifunctional adducts, one with the iminoether ligands in their original E configurations and the other with the iminoether ligands having one *E* and the other, *Z* configurations. The coordination geometry obtained by energy minimization calculations is in qualitative agreement with 2D NMR data.


### ANTITUMOR *TRANS* PLATINUM ADDUCTS OF *GMP* AND *AMP*

Yangzhong Liu[1], Maria F. Sivo[2], Giovanni Natile[2] and Einar Sletten*[1]

[1] Department of Chemistry, University of Bergen, Allegt.41, N-5007 Bergen, Norway
<einar.sletten@kj.uib.no>
[2] Department of Pharmaceutical Chemistry, University of Bari, Via E. Orabona 4, I-70125, Bari Italy

### ABSTRACT

Recently it has been shown that several analogues of the clinically ineffective *trans*-DDP exhibit antitumor activity comparable to that of *cis*-DDP. The present paper describes the binding of antitumor *trans*-[$PtCl_2$(*E*-iminoether)$_2$] (*trans-EE*) to guanosinemonophosphate (GMP) and adenosinemonophosphate (AMP). We have used HPLC and $^1$H and $^{15}$N NMR to characterize the different adducts. In the case of a 1:1 mixture of *trans-EE* and GMP, at an early stage of the reaction, a monofunctional adduct is formed which, subsequently, is partly converted into a monosolvated monofunctional species. After about 70 hours an equilibrium is established between chloro and solvato monofunctional adducts at a ratio of 30/70. In the presence of excess GMP (4:1) the initially formed monofunctional adducts react further to give two bifunctional adducts, one with the iminoether ligands in their original *E* configurations and the other with the iminoether ligands having one *E* and the other, *Z* configurations. The coordination geometry obtained by energy minimization calculations is in qualitative agreement with 2D NMR data.

### INTRODUCTION

The antitumor activity of cis-diamminedichloroplatinum(II) (*cis*-DDP), the most widely used metal-based anticancer drug, has been associated with formation of adducts with DNA (for recent reviews see [1]). Since the *trans* isomer of *cis*-DDP is clinically ineffective it was assumed that a structure-activity relationship exists where only the *cis* geometry is therapeutically active. However, recently, it has been shown that several analogues of *trans*-DDP exhibit antitumor activity comparable to that of *cis*-DDP [2-5]. Of particular interest is a group of iminoether derivatives analogous to *trans*-DDP: *e.g.* *trans*-[$PtCl_2$(iminoether)$_2$] which was shown to be endowed with significant *in vivo* antitumor activity [3,6]. Surprisingly, these compounds were remarkably more cytotoxic than their congener *cis*-[$PtCl_2$(iminoether)$_2$] [2].

In addition to the *cis* and *trans* geometry, platinum-iminoether complexes can have either *E* or *Z* configuration depending on the relative position of the alkoxy group and the N-bonded Pt with respect to the C=N double bond, and this represents another aspect to be considered for the evaluation of structure-activity relationships. The DNA binding mode of *trans-EE* in a cell free medium has been characterized by several biophysical methods [7]. As a result of these studies it has been concluded that kinetically stable *trans-EE* forms monofunctional adducts at guanine residues in double-helical DNA. As a consequence *trans-EE* is assumed to modify DNA differently to clinically ineffective *trans*-DDP.

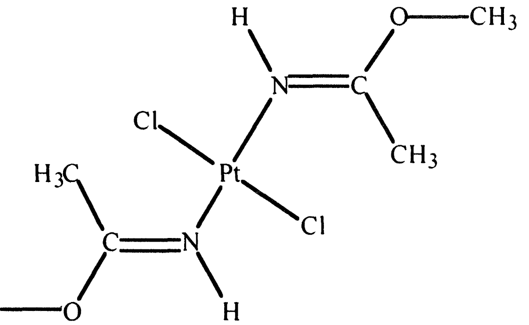

Trans-[$PtCl_2${*E*-HN=C(OMe)Me}$_2$] (*trans-EE*)

The nature of the non-leaving groups, i.e. the amine ligands in *trans*-DDP and the iminoether ligands in *trans-EE*, somehow play an important role and it has been speculated that in the latter case one of the drug - DNA interactions could not involve directly the metal ion but be mediated by the iminoether ligand. Such a type of interaction with DNA would place the platinum-iminoether complex at the junction between *cis*-DDP and non-metallic cytotoxic agents.

Recently, a detailed HPLC and NMR spectroscopic analysis of the DNA duplex consisting of *trans-EE* platinated 5′-d(CCTCG*CTCTC) ($I_M$) and its complement 5′-d(GAGAGCGAGG) (II) was described [8]. The analysis revealed that the binding pattern of *trans-EE* is more complex than first anticipated involving pH and sequence-dependent isomerization reactions and deplatination when the NMR sample contains high NaCl concentration (0.2 M). This paper describes a more detailed picture of the binding pattern between *trans-EE* and mononucleotides (GMP and AMP).

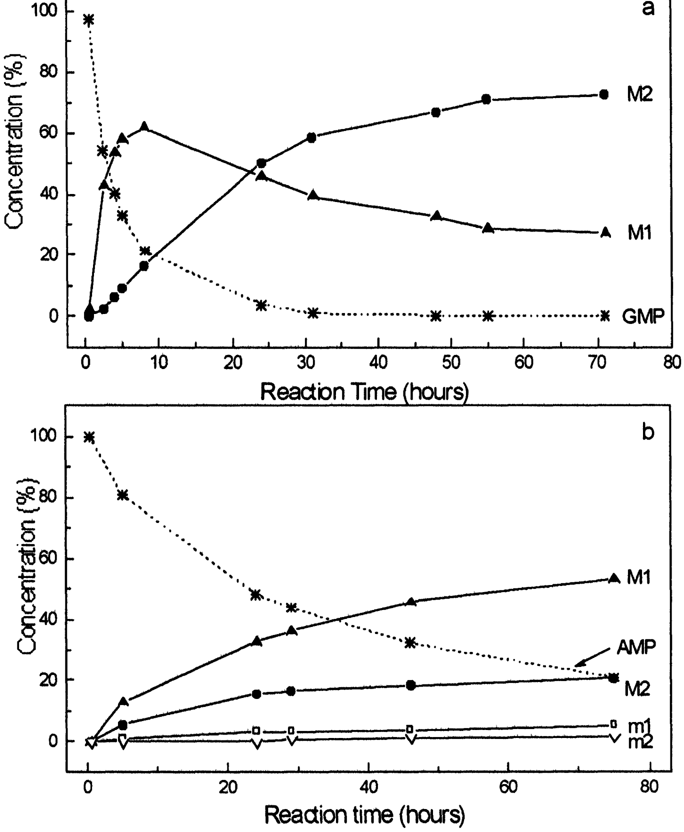

**Figure 1.** Distribution curves for the *trans-EE* platination reaction of nucleotides at 1:1 molar ratio. (a) GMP (✱), (b) AMP. at pH 6.8 in $H_2O$, 298 K.

### MATERIALS AND METHODS

*Sample preparation.*

The *trans-EE* complex was synthesized by the Bari group as previously described [9]. 5′-riboguanosinemonophosphate (sodium salt) (GMP) and the corresponding adenine phosphate (AMP) were purchased from Sigma and used without further purification. Deuterium oxide was purchased from Flurochem Limited Company. The reactions between *trans-EE* and the mononucleotides were performed using the $PtCl_2[E\text{-}HN{=}C(OMe)Me]_2$ and the nucleotide in molar ratios 1:1 and 4:1, respectively, at pH 6.8. The solutions were kept in the dark at room temperature. The platinated nucleotides were purified by HPLC on a Waters 626 LC instrument using the Millenium 32 software [10]. A reverse phase Waters Symmetry C8 column was used with gradient eluent (0-50 % methanol) in 50 mM $NaClO_4$ at a flow rate of 0.8 ml/min. The reactions were monitored by HPLC until the equilibrium was reached. The platinated samples for NMR experiments were separated by HPLC, lyophilized and dissolved into 0.5 ml $D_2O$. No buffer was added and the pH value of the solution was about 4. The samples of unplatinated GMP and AMP were dissolved in 0.5 ml $D_2O$, pH 5.5 (final concentration: 0.1 M).

*NMR Spectroscopy.*

NMR experiments were performed on a Bruker DRX 600 instrument, operating at 600 MHz for $^1H$ NMR spectroscopy. 1D $^1H$ NMR spectra were collected with a total of 32K complex points and 64 transients. For all 1D and 2D spectra the spectral width were 7200 Hz and a relaxation delay of 2 s was used. The presaturation pulse sequence was used for the samples in $D_2O$ and the 3-9-19 WATERGATE pulse sequence was used for the samples in $H_2O$ solution for water suppression [11]. In the ROESY and NOESY experiments, a total of 2048 complex points in $t_2$ were collected for each of 256 $t_1$ increments and 80

transients were averaged for each increment. Two-dimensional [$^1H$, $^{15}N$] HMQC spectra with 1 K complex points along $t_2$ were recorded for 128 transients. The experiment was optimized for two-bond $^1H$-$^{15}N$ connectivity involving nonexchangeable protons, where $\Delta = 1/2J_{NH} = 48$ ms. Coupling constants are found in the range of 6-14 Hz, except for $^2J_{N9-H1'}$ which is found to be 4.1 Hz in GMP. The $^1H$-$^{15}N$ spectra widths were 1929 and 6082 Hz for free GMP and 1912 and 12064 Hz for the *trans-EE* adduct, respectively. The FID's were multiplied by a square-cosine function and zero-filled to 2048 complex points along $t_1$ and $t_2$. The NMR data were processed on a Silicon Graphics INDY workstation using the program FELIX (Biosym) [12] and on a 133 MHz Pentium PC using 1D and 2D WIN-NMR (Bruker) [13]. The one dimensional $^1H$ FID's were multiplied by an exponential window function prior to Fourier transformation. Typically, 0.3 Hz was added to the line widths. No baseline correction was needed. $^1H$ spectra were referenced to the HDO-resonance at 4.76 ppm at 298 K and $^{15}N$ spectra relative to saturated $NH_4Cl$ solution at -352.9 ppm.

### RESULTS

### *HPLC analysis*

Small fractions of the 1:1 *trans-EE*/GMP reaction mixture were analyzed by HPLC at several time intervals. The species distribution is given in Figure 1a. Initially, two species M1 and M2 are formed at different rates, the faster forming species, M1, attains a maximum concentration after ca 10 hours and is then partly converted to a second species, M2; at equilibrium the ratio M1/M2 is *ca.* 30:70. The reaction proceeds with a half-time of 3.5 h (at 298 K, $c_{Pt} = 1$ mM).

For the reaction with excess GMP (4:1) a more complex mixture of products was formed. Similarly to the case of a 1:1 ratio of reactants, two monofunctional adducts M1 and M2 are formed initially and are then converted into two bifunctional species (M3 and M4) which reach a concentration ratio of 5:1 after 20 days and have chromatographic retention times of 34 and 33 min., respectively. For longer reaction time the amount of M4 increased further and after two months exceeded that of M3.

For AMP the reaction is much slower than for GMP (Figure 1b). The half-time $t_{1/2} = 23$ h is estimated from the HPLC trace. After about 7 days about 91 % of AMP has reacted. As for GMP two major species M1(60%) and M2 (20%) are formed, a chloro and an aquacomplex, respectively. In addition, two minor species (less than 5%) are formed, probably representing the corresponding chloro and aqua N1 platinated complexes.

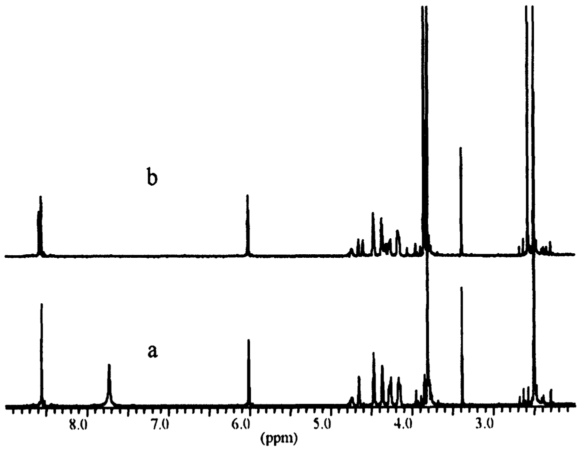

**Figure 2.** $^1H$ NMR spectra for adduct M1 recorded at different time intervals, pH 4.8, in $D_2O$ at 298 K.
(a) newly prepared sample. (b) 20 hours later.
(b) (The peak at 3.4 ppm is the trace residue of methanol from HPLC eluent.)

### NMR assignments

*Monofunctional GMP adducts.* The 1D $^1H$ NMR spectra were recorded at different time intervals for the reaction mixture and for the separate HPLC fractions. Figure 2 exhibits the 1D spectra for M1 recorded at different time intervals. Integration of the 1D spectra confirms the 1:1 ratio of GMP to *trans-EE*, (1:6 of H8 to $CH_3$ or $CH_3O$ peak). The NH proton of the iminoether ligands exchanges slowly with $D_2O$ during 2D experiment at 298 K and disappear after about 20 hours. The proton chemical shifts of the resonance of the different platinated species are listed in Table 1.

The G-H8 resonance for M1 and M2 are shifted 0.46 ppm and 0.40 ppm downfield relative to unplatinated GMP, respectively. Platinum coordination at N7 is usually found to produce H8 downfield shifts in the 0.5 - 1 ppm range as a result of electron-withdrawing effect of the metal. The resonances of the sugar protons exhibit only insignificant chemical shift changes relative to native GMP. Also, the proton chemical shifts of the methyl and the methoxide groups, 2.46 ppm and 3.78 ppm, respectively, are almost identical to those of free *trans-EE* in solution [9].

The two species, M1 and M2, interchange reversibly depending on pH and chloride concentration. The equilibrium ratio M1/M2 = 0.35 at pH 6.8 increases to 0.7 at pH 1.7. Addition of NaCl to the sample also induces a conversion of M2 to M1 indicating that the latter is the chloro complex and M2 the aqua complex. Plots of G-H8 chemical shifts *vs*. pH for M1 and M2 are shown in Figure 3. The $pK_a$ at about 8.5 corresponds to guanine N1 deprotonation. The absence of change in chemical shift at pH < 3 is clearly diagnostic for G-N7 platination. The change of chemical shift at 5.8 corresponds to the influence of phosphate (de)protonation.

Table 1. $^1H$ NMR chemical shifts (ppm) for GMP and *trans-EE* platinated adducts. Reference: HDO signal at 4.76 ppm, 298 K, pH in parentheses.

| δ (ppm) | GMP (7.1) | M1 (4.8) | M1 (6.8) | M2 (4.8) | M2 (6.8) |
|---|---|---|---|---|---|
| H8 | 8.17 | 8.55 | 8.63 | 8.58 | 8.57 |
| H1' | 5.91 | 6.01 | 5.96 | 6.02 | 5.94 |
| H2' | --[a] | 4.65 | 5.96 | 4.60 | 5.94 |
| H3' | 4.46 | 4.47 | 4.46 | 4.46 | 4.45 |
| H4' | 4.30 | 4.36 | 4.34 | 4.37 | 4.33 |
| H5' | 3.98 | 4.27 | 4.07 | 4.32 | 4.05 |
| H5" | 3.98 | 4.15 | 4.09 | 4.15 | 4.03 |
| NH[b] | — | 7.73 | 7.78 | 7.92 | 7.95 |
| $OCH_3$ | — | 3.81 | 3.76 | 3.85 | 3.78 |
| $CH_3$ | — | 2.50 | 2.45 | 2.58 | 2.46 |

a. Overlap with residual HDO at 4.7-4.8 ppm
b. NH protons are slowly exchanged in $D_2O$ during 10-20 hours.

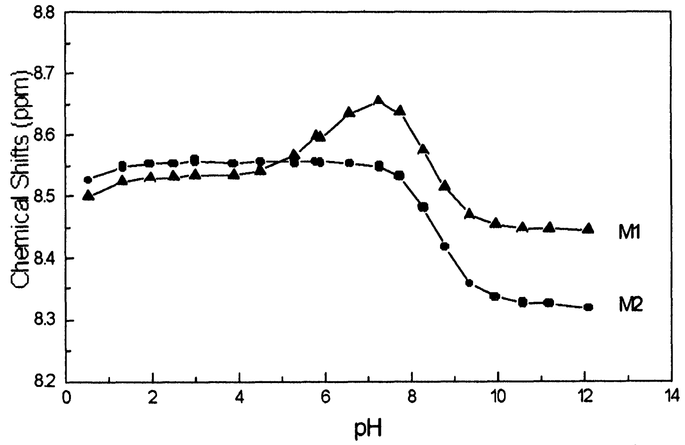

**Figure 3.** Titration curves for G-H8 of adducts M1 and M2. (298 K in 90 % $H_2O$)

The 2D $^1H$ ROESY spectra for both M1 and M2 show a medium size NOE crosspeak between G-H8 and $CH_3$ of *trans-EE.* A large cross peak between H8 and CMe is expected since both are cis to platinum with respect to the C=N double bound and therefore can come very close to each other during rotation of the ligands about the Pt -N bonds. No crosspeak corresponding to G-H8... $CH_3O$ was detected above the noise level. A strong intra ligand crosspeak is observed between the imino proton and the methoxide group confirming that the iminoether ligands retain the configuration also after coordination of GMP.

*Bi-functional adducts.* The bifunctional adducts M3 and M4 were characterised by $^1H$ and $^{15}N$ NMR spectroscopy. The 2:1 ratio of GMP to *trans-EE* ratio was confirmed by integration of H8 and $CH_3$ (1:3 peak intensity). The G-H8 proton chemical shifts for the two isomers are almost identical to those of the M1 and M2 adducts. The main difference between the spectra of M3 and M4 is that the methyl and methoxide groups give rise to one set of signals for M3 but two sets for M4 (Figure 4 a, b).

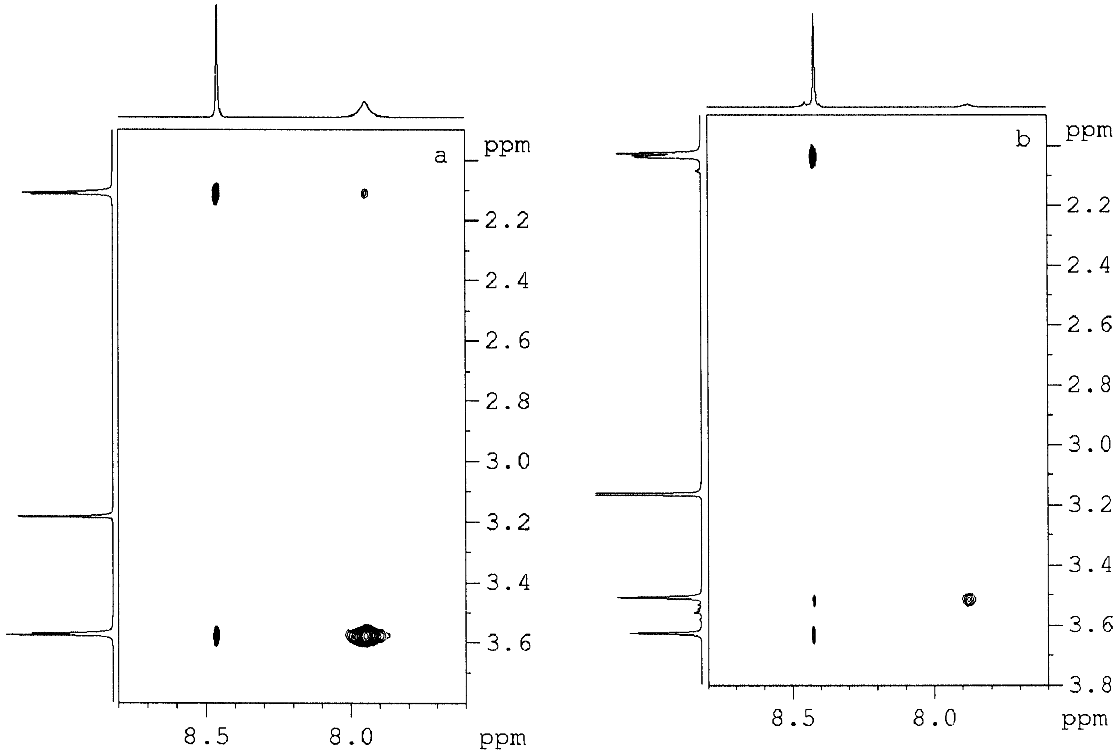

**Figure 4.** Selected regions of the ROESY spectrum of the bifunctional GMP/*trans-EE* adducts showing contacts between G-H8 and the methyl and methoxide groups. The intra *E*-iminoether ligand cross peaks involving OMe and N-H are also apparent. (a) M3, (b) M4

The ROESY spectrum of M3 shows two distinct crosspeaks involving H8, one strong to the methyl group and one less intense to the methoxide group. In addition a strong intra-ligand crosspeak is observed between the methoxide group and the imino proton. In M4 the two partly overlapping methyl signals are both coupled to H8 while the crosspeak between OMe and NH is much greater for the isomerized iminoether (Z configuration) than for the non isomerized one (E configuration)

$^{15}N$ NMR is a sensitive probe for detecting specific nitrogen binding sites on nucleobases. The use of $^{1}H$-detected $^{1}H$-$^{15}N$ HMQC spectroscopy greatly enhances the sensitivity of natural abundance $^{15}N$ spectroscopy. The $^{15}N$ resonances of N7 and N9 are easily observed through multi-bond scalar coupling with G-H8. A cross peak between N9 and the anomeric proton H1′ confirms the assignments. For the *trans-EE*/GMP M3 complex the N7 signal is shifted to -238.4 ppm (97,5 ppm upfield that of free GMP), while N9 exhibits a minor shift to -202.9 ppm (4.3 ppm downfield that of free GMP). The large upfield shift induced by platination is comparable to similar shifts observed for protonation of purine nitrogens (~70 ppm).

*The AMP adduct.* The *trans-EE* binding to AMP follows the same pattern as that observed for GMP. A-H8 is shifted 0.4 ppm downfield while A-H2 is not affected. $^{1}H$-$^{15}N$ HMQC spectroscopy of platinated AMP (M1) shows significant upfield shift of N7 (-247.7 ppm as compared to a free AMP N7 value of -150.8 ppm) while N3 and N9 are only shifted downfield by about 10 ppm. This shift confirms the N7 binding of AMP in *trans-EE* adducts. of M1 at pH 4, N1 is partly protonated, therefore, the N1 signal shifts to high field by 22.3 ppm. ROESY spectra show the AMP adduct to have a relationship to the iminoether ligand similar to GMP. A medium sized ROESY crosspeak is observed for the A-H8 $CH_3$ contact. Another very weak crosspeak is observed for the A-H8 $CH_3O$ contact. In addition, the anomeric proton has weak contacts to the methyl group.

*Molecular modeling.* In the molecular modeling approach using the BIOSYM software [14] for AMBER-minimized energies the *trans-EE* complex was fixed in the geometry adopted in the solid state [9] except for the methyl groups which were replaced by pseudoatoms calculated from averaging the hydrogen X-ray coordinates. The two iminoether ligands are not coplanar in the X-ray structure but are twisted 35° to relieve steric crowding of the methyl groups. In the calculation the configuration of the iminoether ligands were fixed but allowed to rotate freely about the Pt-NH bonds. Also the GMP ligand was allowed to rotate freely around the Pt-N7 bond while keeping the square-planar geometry fixed. Both the monofunctional and the bifunctional models were subjected to energy minimization.

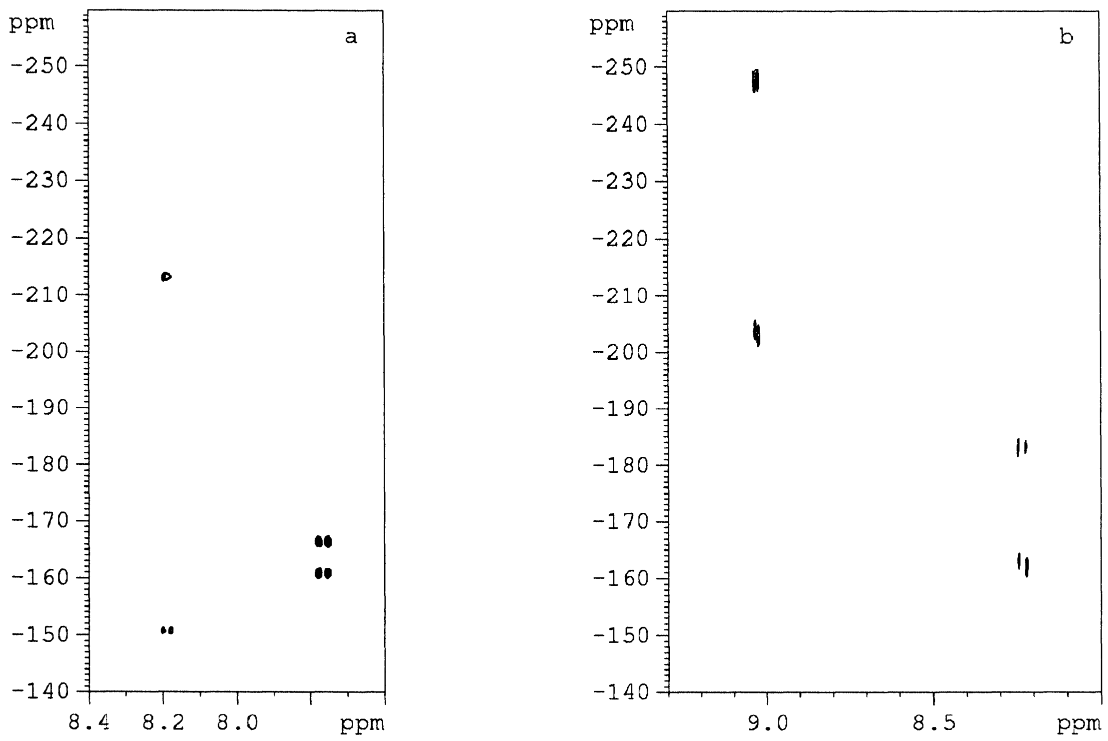

**Figure 5.** $^1H$-$^{15}N$ correlation NMR spectrum at 280 K. (a) AMP, pH 7, (b) *trans-EE* adduct M1, pH 4.

No symmetry constraints were applied to the relative positions of the chemically equivalent GMP ligands. The computational details are given in ref. [8]. One may add that most probably there exixts a distribution of several conformers. However, the NMR spectra were recorded at a temperature at which the signals from different conformers are not resolved. A stereo-view of the refined molecular model is shown in Figure 6. Inter and intra through-space H-H contacts are in qualitative agreement with the NMR data. The H8···$CH_3$ distance of 3.47 - 3.70 Å is in agreement with a relatively strong cross peak while the H8···$CH_3O$ distance of 4.49-4.93 Å gives a comparatively weak cross peak.

### DISCUSSION

Previously we have reported on the NMR structure of an oligonucleotide duplex platinated by *trans-EE* at a guanine N7 site [8]. Only the major monofunctional adduct was structurally characterized, however, two minor species were also present as evidenced by HPLC analysis. In this context it is of interest to explore the relation between monofunctional *vs* bifunctional adduct formation which is closely related to the ability to engage in inter and intra strand cross-linking reactions. Other *trans*-Pt complexes with bulky ligands, *e.g.* pyridine, have been reported to have considerable enhanced ability to form interstrand cross-links over both its *cis* isomer and the $NH_3$ complexes [15]. This finding is contrary to the situation for DNA incubated with *trans-EE* for 48 hr at 37 °C in 10 mM $NaClO_4$ which was shown to produce only marginal amounts of interstrand cross links [16]. Bierback and Farell [17] also showed that this reaction is drastically slowed ($t_{1/2}$= 17 h, T = 310 K) when one of the ammine ligands was replaced by quinoline. This latter compound exhibits the same tendency as *trans-EE* to form long-lived monofunctional adducts on DNA, clearly different from the behavior of *trans*-DDP.

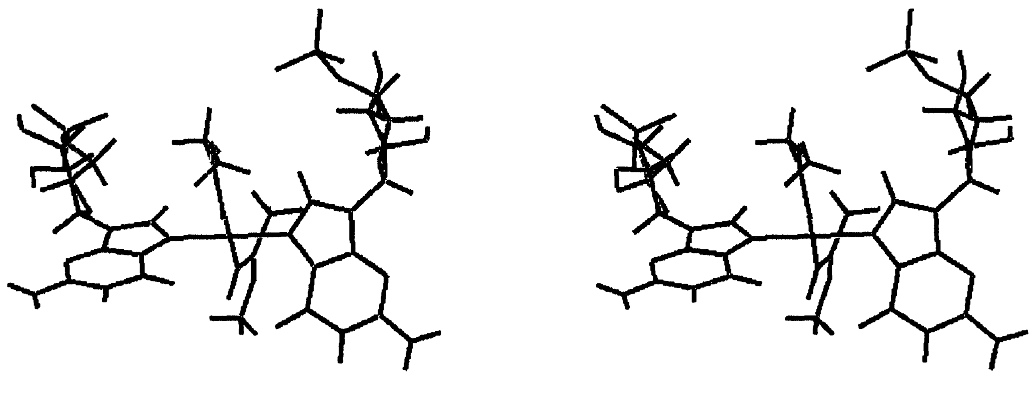

**Figure 6.** Stereoview of the energy minimized molecular model of the bifunctional GMP/*trans-EE* adducts.

Comparison of reaction rates for *trans-EE* platination of mononucleotides and oligonucleotides indicates that the *trans-EE* platination of the G-residue in the single strand oligonucleotide d(CCTCGTCTC) proceeds at about the same rate as for the mononucleotide (Figure 7) [8]. For double helical DNA the geometric restraints may drastically reduce the *trans-EE* platination rate.

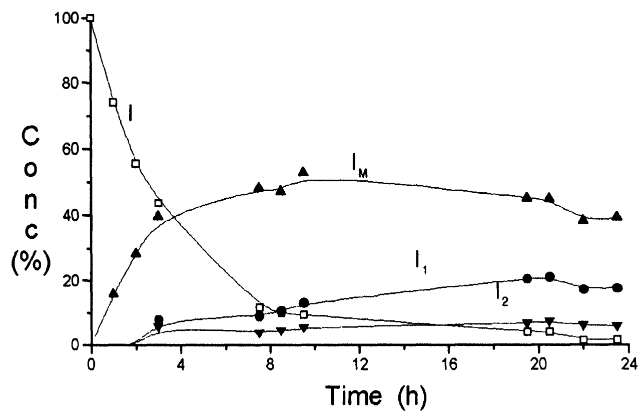

**Figure 7.** Species distribution curves for *trans-EE* platination of d(CCTCGCTCTC): (▲) Major adduct ($I_M$), (●) and (▼) Minor adducts, (❍) non-platinated oligonucleotide. pH 2.5, no salt, at room temperature in $H_2O$. (From ref. [8]).

The difference between the GMP adducts M1 and M2 is deduced from monitoring the equilibrium as a function of pH and addition of NaCl. Most likely M1 represents the GMP chloro complex and M2 the corresponding solvato species. From the ROESY spectra it is evident that, in both M1 and M2, there are strong crosspeaks between H8 of GMP and the C-Me groups of the iminoether ligands which come very close during rotation of the ligands about the Pt-N bonds.

With excess GMP (4:1) the initially formed monofunctional adducts are converted to bifunctional *trans-EE*$(GMP)_2$ adducts (M3 and M4). The transition is quite slow and takes approximately 15 days to be completed at 298 K and pH 6.8. The retention time for M3 and M4 are surprisingly close to the retention time for the monofunctional adducts. The M3 bifunctional adduct has preserved the configuration of the iminoether ligand (*E*) while in the M4 bifunctional adduct one iminoether ligand has isomerized to the Z configuration. The concentration of the M4 isomer is initially only 1/5 of M3, however, after two months the ratio has increased to approximately 1:1. This implies that M4 has similar stability to that of M3. This is a rather unexpected result since in the starting dichloro species the isomer with both iminoether ligands in the *E* configuration is far more stable than any other isomer. Therefore it appears that the ancillary ligands (the purine bases in this case) are able to influence the thermodynamic stability of the iminoether configurations. Similar geometry conversion processes were found in nucleoside-*trans-EE* adducts (unpublished results).

ACKNOWLEDGMENTS. Financial support by the European Commission BIOMEDII program (Contract BMH4-CT97-2485) and COST action D8/007/97 and D8/012/97 are gratefully acknowledged. Thanks are extended to the Norwegian Research Council for financial support (Contract 135055/410) and the Italian M.U.R.S.T.(Cofin 1988 n° 9803021072).

### REFERENCES

1. B. Lippert (ed.) *Cisplatin. Chemistry and Biochemistry of a Leading Anticancer Drug.* Wiley-VCH, Zurich, 1999.
2. G. Natile, M. Coluccia, in: *Topics in Biological Inorganic Chemistry, Metallopharmaceuticals I, DNA Interactions.* **1**, 73-98, (1999) (M. J. Clarke, P. J. Sadler, eds). Springer-Verlag, Berlin.
3. M. Coluccia, A. Nassi, F. Loseto, A. Boccarelli, M.A. Mariggio, D. Giordano, F.P. Intini, P. Caputo, G. Natile. *J. Med. Chem.* (1993) **36**, 510-512.
4. N. Farrell, in: *Metal Ions in Biological Systems.* A. Sigel and H. Sigel (eds.) (1996) **32**, 603-639. Marcel Dekker, Inc., New York. N. Farrell, L.R. Kelland, J.D. Roberts, M. Van Beusichem. *Cancer Res.* (1992) **52**, 5065-5072.
5. L.R. Kelland, C.F.J. Barnard, I.G. Evans, B.A. Murrer, B.R.C. Theobald, Wyer, S.B., P.M. Goddard, M. Jones, M. Valenti, A. Bryant, P.M. Rogers, K.R. Harrap. *J. Med. Chem.* (1995) **38**, 3016-3024.
6. M. Coluccia, A. Boccarelli, M.A. Mariggio, N. Cardellicchio, P. Caputo, F.P. Intini, G. Natile. *Chem. Biolog. Interactions,* (1995), **98**, 251-266.
7. R. Zaludova, G. Natile, V. Brabec. *Anti-Cancer Drug Design* (1997), **12**, 295-309.
8. B. Andersen, N. Margiotta, M. Coluccia, G. Natile, E. Sletten. *Metal-Based Drugs* (2000) **7**, 23-32.
9. R. Cini, P.A. Caputo, F.P. Intini, G. Natile. *Inorg. Chem.* (1995) **34**, 1130-1137.
10. Waters Millenium 32 software.
11. V. Sklenar, M. Piotto, R. Leppik, V. Saudek. *J. Magn. Reson. A* (1993) **102**, 241-245.
12. FELIX (version 2.30) Processing package, BIOSYM/MSI.
13. Bruker 1D WIN-NMR (version 960901.2) and 2D WIN-NMR (version 6.02).
14. BIOSYM User Guide for Insight II (version 2.3.5), Discover (version 2.9.5).
15. Y. Zou, B. Van Houten, N. Farell. *Biochemistry* (1993) **32**, 9632-9638.
16. V. Brabec, O. Vrana, O. Novakova, V. Kleinwachter, F.P. Intini, M. Coluccia, G. Natile. *Nucleic Acids Res.* (1996) **24**, 336-341.
17. U. Bierbach and N. Farell. *Inorg. Chem.* (1997) **36**, 3657-3665.

**Received: August 16, 2000- Accepted: September 11, 2000 - Received in revised camera-ready format: September 14, 2000**

